# Cost Effectiveness Analysis of Avonex and CinnoVex in Relapsing Remitting MS

**DOI:** 10.5539/gjhs.v7n2p139

**Published:** 2014-10-08

**Authors:** Behzad Najafi, Hossein Ghaderi, Mehdi Jafari, Smaeil Najafi, Aliasghar Ahmad Kiadaliri

**Affiliations:** 1 Health management and economic research center, School of Health Management and Information Sciences, IUMS, Tehran, Iran; 2 Department of Health Economics, School of Health Management and Information Sciences, IUMS, Tehran, Iran; 3 Research Center for Health Services Management, Institute for Futures Studies in Health, Kerman University of Medical Sciences, Kerman, Iran; 4 Department of Health Management and Economics, School of Public Health, Tehran University of Medical Sciences, Tehran, Iran

**Keywords:** multiple sclerosis, cost-effectiveness, CinnoVex, HRQoL, Avonex

## Abstract

**Introduction:**

Multiple sclerosis is a chronic and degenerative neurological disease characterized by loss of myelin sheath of some neurons in brain and spinal cord. It is associated with high economic burden due to premature deaths and high occurrence of disabilities. The aim of the current study was to determine cost effectiveness of two major products of interferon 1a in patients with relapsing-remitting multiple sclerosis.

**Method and Materials:**

Altogether, 140 patients who have consumed Avonex and CinnoVex in Relapsing Remitting MS for at least two years were randomly selected (70 patients in each group). Health-related quality of life (HRQoL) was assessed using the adopted MSQoL-54 instrument. Costs were measured and valued from Ministry of Health and Medical Education (MOHME) perspective. Two-way sensitivity analysis was used to check robustness of the results.

**Results:**

Patients in CinnoVex group reported significantly higher scores in both physical (69.5 vs. 50.9, P<0.001) and mental (63.3 vs. 56.6, P=0.03) aspects of HRQoL than Avonex group. On the other hand, annual cost of CinnoVex and Avonex were 2410 US$ and 4515US$ per patient, respectively (P<0.001).

**Conclusions:**

The results showed that CinnoVex was dominant option over the study period. It is suggested that results of the current study should be considered in allocating resources to MS treatments in Iran. Of course, our findings should be interpreted with caution duo to short term horizon and lack of HRQoL scores at baseline (before the intervention).

## 1. Introduction

Multiple sclerosis (MS) is a chronic and degenerative neurological disease occurred by loss of myelin sheath of some neurons in brain and spinal cord (Gani et al., 2008; Bell et al., 2007). MS can lead to both physical and cognitive disabilities (Bobholz & Rao, 2003) and its main outcome is progressive inability that leads to low health-related quality of life (HRQoL), and premature deaths (Gani et al., 2008). The main symptoms of MS are fatigue, muscle symptoms which leads to lowly mobility, obstructions in the bowel and bladder, visual symptoms such as inflammation of eyes, pain, changes in cognitive function, sensory disorders, and depression (O’Connor, 2002; Tan, Yu, Tabby, Devries, & Singer, 2010).

There are three types of MS: Primary Progressive MS (PPMS), Relapsing Remitting MS (RRMS) and Secondary Progressive MS (SPMS) (Kremenchutzky et al., 1999). In fact, RRMS is the most common form of MS in both children and adults, and it followed by the secondary and primary progressive forms (Ozakbas, Kaya, & Idiman, 2012). In the most cases, MS begins in early of adolescence (Noonan, Kathman, & White, 2002; Confavreux, Aimard, & Devic, 1980) and two-thirds of patients are 20-30 years old on their MS onset. The incidence of MS in younger is low and the onset of disease among people younger than 16 years is about 1.2–6% (Ozakbas, Idiman, Baklan, & Yulug, 2003; Simone et al., 2002). A recent study in Iran reported that 70.8% of patients were 20–40 years at the time of the first episode of neurological impairments (Rezaali, Khalilnezhad, Naser Moghadasi, Chaibakhsh, & Sahraian, 2013). Peak age of onset in women is 5 years higher than men. Approximately in 10% of cases MS begins before 18 years old. While the prevalence of MS is high in North Europe, North America and Australia; it is generally a rare disease in Asia. It is estimated that there are 10–15 cases per 100 000 populations in Iran, while there are 30–80 cases per 100 000 populations in Europe. More recently, an epidemiological study of MS in the southeastern part of Iran, found that the incidence rate of MS has a faster growth rate than what was estimated in previous years (Rezaali, Khalilnezhad, Naser Moghadasi, Chaibakhsh, & Sahraian, 2013).

Although MS has been identified many years ago but yet there is no effective treatment for it. Today a vast range of drugs are used for prevention and treatment of MS and the recombinant Beta interferon is the most common therapeutic option for MS (Shahkarami, Vaziri, Salami, Harandi, & Oger, 2013).

Avonex is one of the main interferon and is used as 30 µg (IM) weekly (Sharafaddinzadeh, Majdinasab, Ghiasian, & Moravej-Aleali, 2011). Several studies showed that it is effective in preventing relapse and reduction in disability and MRI lesions (Etemadifar, Janghorbani, & Shaygannejad, 2006). CinnoVex is a new alternative produced in Iran in recent years and approved by Iranian Food and Drug Organization (IFDA) (Abolfazli et al., 2012) and satisfactory results are achieved in IFDA Safety and Toxicology tests (Sharafaddinzadeh, Majdinasab, Ghiasian, & Moravej-Aleali, 2011).

CinnoVex is a recombinant protein consisting of 166 amino acids with a weight of 22.5 kDa and is produced by Chinese hamster ovary cells. CinnoVex is bio similar (the production process and quality control is the same) with Avonex exactly (Etemadifar et al., 2010).

Studies showed that CinnoVex prevents the progression of disability in MS patients (Abolfazli et al., 2012). Moreover, it controls relapsing and re-attacks of MS. likewise other drugs, consuming of CinnoVex is associated with a number of side effects (Etemadifar et al., 2010). However, there aren’t significant differences in side effects between Avonex and CinnoVex and both are the same in quality (Abolfazli et al., 2012).

Due to its progressive and chronic nature, MS incur a significant economic burden, especially considering that it affects people in their productive years of life (Bell et al., 2007). The estimation show that economic burden of MS in Germany, Italy, Sweden and the UK was equal to 4252, 1613, 541 and 2708 million Euros in 2003, respectively (Kobelt, 2006). Previous studies in Europe and USA showed that costs of preventive medicine are substantial (e.g., it was more than US$16,000 per patient in USA (Lundy & Craig, 2006; Henriksson, Fredrikson, Masterman, & Jonsson, 2001) and US$45,000 in Sweden (Kobelt, 2006). However, as progressing to more severe status of the disease lead to higher costs, it is believed that preventive programs in relapsing of disease might reduce resources utilization and thereby offset their costs (Grima et al., 2000; O’Brien, Ward, Patrick, & Caro, 2003). A recent study showed that disease- modifying drugs can result in substantial medical and indirect cost savings (Birnbaum et al., 2009).

Cost-utility and Cost-effectiveness analyses are two useful tools in economic evaluation for assessing the costs and benefits of alternative treatment options. Limited health resources imply that economic evaluation analysis can aid informed decision making (Orlewska et al., 2005).

## 2. Methods and Materials

### 2.1 Patients and Treatments

In a retrospective chart review study, we collected list of all patients on CinnoVex (n=2,300) and Avonex (n=520) treatments in three clinical centers in Tehran. We selected our sample among patients who were 22 years and older and have consumed the drug for last two years and longer. Based on Chilcott et al. (Chilcott, Mccable, Tappenden, & Abrams, 2003) and assuming α= 0.05, β=0.02, P1=0.2, and P2=0.41 in following formula, we calculated a sample size of 70 patients in each treatment arm (i.e., 140 patients were randomly selected). In any case, if a patient did not consent to participate, the next patient from the list was contacted to participate.


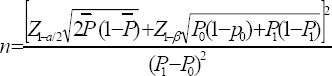


### 2.2 Outcome Measurement

We used HRQoL as outcome measure. HRQoL was measured using Multiple Sclerosis Quality of Life (MSQoL54) instrument.

MSQOL-54 is a questionnaire consists of a well-validated generic health status tool. It consists of short form36-item (SF36) health survey questionnaire and 18 extra items which are MS-specific (Ware, 1992). There are eight distinct domains in the SF-36 including *physical function* (10 questions) that apprise activities might do during a typical day, *social functions* (3 questions) investigate about social activities, *energy* (5 questions) evaluates energy and feeling tired, *physical roles limitation* (4 questions) ask about any problems in working or other regular daily activities as a result of physical health, *emotional roles limitation* (3 questions) apprise about any problems in working and other regular daily activities as a result of emotional problem such as feeling depressed or anxious, *pain* (3 questions) seek about the bodily pain, *emotional wellbeing* (5 questions) ask about happiness and calm and peaceful, and *general health* (6 questions) ask about the general health perception.

Three items were added to these domains (i.e., social function, pain and energy) and the remaining 15 novel items constitute new domains including health distress (4questions) ask general question such as weighed down by health problems, frustrating about health and discouraging by health problems, sexual function and satisfaction with sexual function (5 questions), cognitive function (4 questions) covers problem in concentrating and thinking and overall QoL (3 questions) (Parkin et al., 1998). The additional items of MSQOL-54 were built based on clinicians and nurse specialist thoughts, without any using of patients viewpoints (Parkin et al., 1998).

MSQoL-54 questionnaire evaluates health condition in two main aspects including physical and mental health. The physical health covers 8 domains and is measured by 39 questions. Mental health contains 5 domains and is measured by remaining 15 questions (Barbara & Vickrey, 1995). In the current study; the questionnaires were filled by direct (face to face) interview.

### 2.3 Cost-Measurement

Costs were measured and valued from third-party payer perspective that in the current study includes the Iranian Ministry of Health and Medical Education (MOHME). As data were gathered retrospectively, we only included the amount of consumed drug in our analysis. In addition, based on current protocol, other costs including diagnosis tests are the same for these treatments (Sharafaddinzadeh, Majdinasab, Ghiasian & Moravej-Aleali, 2011; Sahraeiyan, 2008). Furthermore, a previous study showed that there are no significant differences in side effects of these two treatments (Sharafaddinzadeh, Majdinasab, Ghiasian & Moravej-Aleali, 2011). To calculate cost, the consumed drugs were multiplied in drug prices obtained from Iranian Food and Drugs Association and MS Society. Total cost was calculated for one year and Iranian Central Bank exchange rate was used for exchanging to USD (1USD=12,260 Rial).

### 2.4 Sensitivity Analysis

A two-way sensitivity analysis was performed to assess the robustness of the study results. To select the changes in cost and HRQoL, we consult with food and drug association experts and clinicians. Based on these, 5% increase in cost and 10% increase at HRQoL in CinnoVex group were applied.

## 3. Results

### 3.1 Descriptive

Table 1 displays the patients’ characteristics in both treatment arms. The mean age of respondents was 36.75 year, and67% were women. The mean duration of drug consumption was 8.3 (± 0.33) and 3.81 (± 0.21) in Avonex and CinnoVex groups, respectively (*p*<0.001).

**Table 1:** Demographic characteristics of patients

		Avonex	CinnoVex	*P*-value	% of all
Gender					
	Male (%)	35	32		33
	Female (%)	65	68		67
marital					
	Marriage (%)	61	68		65
	Single (%)	39	32		35
Age(years)		37.43	36.12	0.000	-
Time of drug consumption		8.3±0.33	3.81±0.21	0.000	-
Weight		68.2	70.01	0.000	-

*Source:* Authors’ results.

### 3.2 Quality of Life

Table 2 shows the crude scores in each scales of MSQoL-54 for both groups.

**Table 2:** Mean of MSQoL_54 in Avonex and CinnoVex

MSQoL_54 Scales	Avonex	CinnoVex	*p* value
Physical function	40.5±14	67.8±14.2	0.000
Health perceptions	58.9±15	66.8±13.2	0.001
Energy/fatigue	40.7±12.1	57.1±11.6	0.000
Role limitations - physical	28.2±9	65.6±15.1	0.000
Pain	67.2±10.2	81.1±14.5	0.000
Sexual function	55.6±12.4	60.1±16.3	0.067
Social function	60.6±11.7	76.3±9.5	0.000
Health distress	59.8±10.1	82±13.3	0.000
Overall quality of life	68±12.6	73±10.9	0.012
Emotional well-being	53.5±13.7	58.1±15.6	0.064
Role limitations- emotional	49.3±9.4	52±11.3	0.124
Cognitive function	57.7±13	62.6±12.1	0.021

It can be seen that the patients in CinnoVex arm had higher scores in all scales than Avonex group and these were statistically significant in 9 out of 12 scales of MSQoL-54 (*p*<0.05). The highest difference between two groups was observed in the physical role limitation scale. On the other hand, there was no significant difference between two groups in the emotional role limitation domain.

Table 3 shows weighted scores of MSQOL-54 scales and its two composite domains (i.e., physical and mental). The patients in CinnoVex group reported significantly higher scores in both physical and mental health composite domains than did patients in Avonex group.

**Table 3:** The mean of MSQOL-54 values and final HRQoL in CinnoVex and Avonex groups

MSQOL-54 Scale	Weight*	Avonex		CinnoVex	

		scale value	outcome	scale value	outcome
Physical function	0.17	40.5	6.9	67.8	11.5
Health perceptions	0.17	58.9	10	66.8	11.4
Energy/fatigue	0.12	40.7	4.9	57.1	6.9
Role limitations - physical	0.12	28.2	3.4	65.6	7.9
Pain	0.11	67.2	7.4	81.1	8.9
Sexual function	0.08	55.6	4.4	60.1	4.8
Social function	0.12	60.6	7.3	76.3	9.2
Health distress-physical	0.11	59.8	6.6	82	9

**Physical Health Composite**			50.9		69.5

***p* value = 0.000**					

Health distress-mental	0.14**	59.8	8.4	82	11.5
Overall quality of life	0.18	68	12.2	73	13.1
Emotional well-being	0.29	53.5	15.5	58.1	16.8
Role limitations – emotional	0.24	49.3	11.8	52	12.5
Cognitive function	0.15	57.7	8.7	62.6	9.4

**Mental Health Composite**			56.6		63.3

***p* value = 0.032**					

*. Weights from Table 2 in Multiple Sclerosis Quality of Life (MSQoL)_54 instrument (Barbara & Vickrey, 1995).

**. Weights from Table 3 in Multiple Sclerosis Quality of Life (MSQoL)_54 instrument (Barbara &Vickrey, 1995).

### 3.3 Costs

We computed costs in our cost-effectiveness analysis from the MOHME perspective, but to provide more detailed data, we reported the drug costs from other financer perspectives in both groups.

Based on benefit package for MS patients, Avonex and CinnoVex are prescribed as 30 μg intra muscular weekly (Sahraeiyan, 2008). The price of each 30μg Avonex vials is 2,200 thousand Iranian Rial (179$). The 50% of this price is subsidized by government. Therefore the consumer price is 1.100 thousand Rial (90$). This means that pharmacies should supply it in 1,100 thousand Iranian Rial. Based on Iranian health insurance regulation on special diseases (Note 1), heath insurance covers 90% of the outpatient costs and 100% of hospitalization costs. However, health insurance organizations pay 360 thousand Rial (29$) instead of 990 thousand Rial. On the other hand, if a drug has been the best alternative and it is produced and supplied in the country, the health insurance organizations cover 90% of cost of interior product for each similar drug that are imported. This implies that the health insurance organizations pay 90% of CinnoVex’s cost for Avonex users and remain should be paid by patients (29 US$ of 179 US$). Table 4 shows total costs of CinnoVex and Avonex drugs and the share of different agents in financing.

**Table 4:** The share of financial agents in financing of Avonex and CinnoVex

Costs	CinnoVex(USD)*	Avonex (USD)*	Difference
Drugs cost	80**	179	99
Subsidized by government (Minstery of Health)	48	90	42
Health insurance cost	29	29	0
Patient cost	3	60	57

**Annual cost of a patient**			

Annual consume (per patient)	52 (vial)	52 (vial)	0
Total cost of a patient	170	3139	2969
Total cost of government (Minstery of health)	2410	4518	2108
Total cost of Health insurances	1527	1527	0

Total cost	4106	9184	5077

*Source:* authors’ calculation.

*. Iranian Food and Drug Association in 2012.

**. Iranian Central Bank – 2012 (1USD=12 260 IR Rial).

Total costs of each Avonex vial is 2,200,000 Rial (179$) and about 50% of this cost is financed by the MOHME. This figure for each CinnoVex vial is 986, 667 Rial (80$) and 60% of this cost is paid by the MOHME. The health insurance organizations cover the same share for CinnoVex and Avonex.

From MOHME perspective, annual cost of CinnoVex and Avonex are 2,610USD and 4,518US per patient, respectively. Therefore the MOHME pays annually 2,108USD more per patient for individuals consuming Avonex. Moreover, patients who consume Avonex also pay a higher price of 2969 USD compared with people on Cinovex therapy.

### 3.4 Incremental Cost-Effectiveness Ratio (ICER)

The results showed that CinnoVex was less expensive and more effective than Avonex over the study period. This implies that CinnoVex is a dominant option and there is no need to calculate the ICER.

### 3.5 Sensitivity Analysis

The results of the two-way sensitivity analysis showed that CinnoVex was still a dominant option compared to Avonex. This confirms that our finding was robust to changes in costs and HRQoL data.

## 4. Discussion

In this study, we aimed to analyze costs and effectiveness of Avonex and CinnoVex in the patients with RRMS in Iran. We selected two groups of RRMS patients who have consumed these brands of betaferon. We matched all characteristics of two groups as possible. Although the mean duration of drug consummation were different in two groups (*P*<0.001), but as we included only the patients who have consumed the drugs at least two years and over, this cannot affect the outcome based on our consultancy with neurologists.

We found that the patients in Cinovex group reported higher HRQoL than Avonex group in both physical and mental domains. This difference was more profound in physical health domain. In terms of costs and from the MOHME perspective, we found that CinnoVex was cheaper than Avonex. In addition, Cinovex incurred less expense on patients’ than Avonex. However, these results should be interpreted with caution because of some limitations in our study. We did not have the baseline HRQoL in patients and as there were no significant differences in demographic and clinical characteristics between two groups we assumed that it was the same at baseline. In addition, the time horizon in our study was short and only drugs costs were included and this might bias our results and limit generalizability of them. However, it should be noted that drug acquisition costs constitute around 86%-93% of total costs for MS treatment (Imani & Golestani, 2012). In addition, other studies (Sharafaddinzadeh, Majdinasab, Ghiasian & Moravej-Aleali, 2011; Etemadifar et al., 2010) showed that there are no significant differences between these two treatments in term of side effect costs. Another issue that should be considered is exchange rate. Avonex is an imported drug and therefore while the exchange rate has been changed, the price of Avonex is fluctuated.

There are plenty of studies which have evaluated cost-effectiveness of alternative treatments among patients with MS. However, the results are discrepant even in the same context. Several studies reported that Avonex is the cost-effective option compared with other therapies (Imani & Golestani, 2012; Iskedjian et al., 2005), while other studies either did not find any difference in cost-effectiveness (Jankovic et al., 2009) or reported that Avonex is not a cost-effective alternative. Janković et al. showed that in the USA health system, the interferon beta-1a is the most favorable treatment option compared to interferon beta-1b and glatiramer acetate. They also found that symptom management with intramuscular interferon β-1a, subcutaneous glatiramer acetate, subcutaneous interferon β-1a, or intramuscular interferon β-1b may not be cost-effective in a Balkan country regardless of the type of therapy (Jankovic et al., 2009). Furthermore, it was shown that immunomodulatory therapy did not provide any improvement in HRQoL compared with no treatment (Bell et al., 2007). A systematic review of cost-effectiveness studies has also shown that the ICER of these drugs in European countries is very high and they might not be cost-effective (Jankovic et al., 2009).

However, to our knowledge, there are very few studies that compared cost-effectiveness of CinnoVex with other treatment options. Two previous studies found that CinnoVex is a more cost-effective form of IFNB-1a in comparison with Avonex (Abolfazli et al., 2012; Nikfar et al., 2013). Nikfar et al. compared the cost-effectiveness of all brand INF β products for managing patients with RRMS from a societal perspective. They found that, based on suggested threshold for developing countries, all brand INF β products but Avonex are cost-effective in Iran. Similar to our findings, they reported that the best strategy among INF β therapies was CinnoVex (Nikfar et al., 2013). Another study showed the similar results in comparison of CinnoVex and Avonex based on the appearance of neutralizing antibodies (NAbs) in MS patients in Iran (Shahkarami, Vaziri, Salami, Harandi & Oger, 2013). Imani and Golestani evaluated costs and utilities of four disease modifying drugs (Avonex, Betaferon, Rebif and CinnoVex) in relapsing-remitting multiple sclerosis in Iran. Their results showed that in long-term, patients who were treated with Avonex, would gain overall greater benefits compared with patients treated with other DMDs (Imani & Golestani, 2012). While, both these studies applied a life time horizo, the applied perspective were different (i.e., Imani and Golestani applied the Iranian healthcare perspectives, while Nikfar et al. applied a societal perspective). Duo to shorter term horizon, different exchange rate, different perspective, and difference in included costs, our results are not directly comparable with these previous Iranian studies.

## 5. Conclusion and Recommendations

In the current study, we found that the CinnoVex is a dominant (more effective and less expensive) option compared with the Avonex in a sample of MS patients in Tehran, Iran. This finding is in line with previous national studies (Abolfazli et al., 2012; Nikfar et al., 2013). In order to cost saving, it is recommend that policy makers involve patients and physician in decision-making regarding consumption and prescription of different drugs. Comparing cost-effectiveness of the same treatment options (i.e., CinnoVex vs. Avonex) alongside randomized controlled trials and also using modeling approach are topics for future research. It is suggested that the results of the current study should be applied in making decisions regarding resources allocations to MS disease in Iran.
